# Supplementary material for: Acceptability of self-sampling for high-risk HPV DNA testing for primary cervical cancer screening among women in Thyolo, Malawi: A qualitative study
Source: PLOS Glob Public Health. 2025 Sep 24;5(9):e0004763. doi: 10.1371/journal.pgph.0004763 (PMC12459778; doi:10.1371/journal.pgph.0004763)
Supplement: Attachment — Submitted filename: Acceptability of SS.docx [file pgph.0004763.s002.docx]

**Paper Review: Acceptability of self-sampling for high-risk HPV DNA testing for primary cervical cancer screening among women in Malawi**

I commend the authors for their efforts in this manuscript. The manuscript covers the acceptability of self-sampling for high-risk HPV DNA testing for primary cervical cancer screening among women in Malawi. The title is accurate, the abstract is well-written, and the manuscript is well-structured overall. However, there are a few observations I will like the authors to address.

Background: Additional and recent references on the prevalence and incidence of cervical cancer worldwide and in Malawi should be included. The focus should be on the general population, since the study was conducted among the general population of women and not women living with HIV (Line 81).

Methods: The methods section should be more structured to enhance readability; the study design, sampling technique, sample size, etc should come first before the intervention. The data management and analysis plan, and ethical considerations should end the section. Provide a justification for the sample size e,g. was saturation attained at the 10^th^ participant? In addition, was a sampling matrix prepared as an inclusion criteria before recruiting study participants? Also, include information about incentive if any given to the participants. What other permissions and approvals were obtained? The abbreviation “TFA” should be defined at first mention (Line 159). Why was ethicality and opportunity cost excluded from the analysis? (Line 225). Supporting documents should include responses from the COREQ checklist (Line 234).

Result: The ages of the participants looks like they were randomly selected and not purposive. What was the criteria for the purposive selection of the study participants (Table 2)? Please note that manuscript writing is not a dissertation. Hence, the characteristics of the study participants can be shortened and referred to table 2. Avoid repeating points. Privacy has been explained in the earlier paragraph. If there is no direct quote for comfort, consider merging privacy and comfort as one (Line 287-294).

You can focus on the quote directly by excluding “in addition” (Line 303). I suggest that a conceptual framework be drawn to link all the factors in the TFA construct identified in the study. Line 310-311 has already highlighted time as a burden. It is again raised in the next paragraph, Line 320-323. Use the past tense of perceive (Line 410).

Discussion: For a more robust discussion, consider including the following references:

1. Bakiewicz A, Rasch V, Mwaiselage J, Linde DS. “the best thing is that you are doing it for yourself” - Perspectives on acceptability and feasibility of HPV self-sampling among cervical cancer screening clients in Tanzania: A qualitative pilot study. BMC Womens Health. 2020;20(1):1–9.
2. Sormani J, Kenfack B, Wisniak A, Datchoua AM, Makajio SL, Schmidt NC, et al. Exploring factors associated with patients who prefer clinician-sampling to HPV self-sampling: A study conducted in a low-resource setting. Int J Environ Res Public Health. 2022;19(1):1–11.
3. Saidu R, Moodley J, Tergas A, Momberg M, Boa R, Wright T, et al. South african women’s perspectives on self-sampling for cervical cancer screening: A mixed-methods study. South African Med J. 2019;109(1):47–52.
4. Pierz AJ, Ajeh R, Fuhngwa N, Nasah J, Dzudie A, Nkeng R, et al. Acceptability of Self-Sampling for Cervical Cancer Screening Among Women Living With HIV and HIV-Negative Women in Limbé, Cameroon. Front Reprod Heal. 2021;2(January):1–8.
