# Supplementary material for: Acceptability of self-sampling for high-risk HPV DNA testing for primary cervical cancer screening among women in Thyolo, Malawi: A qualitative study
Source: PLOS Glob Public Health. 2025 Sep 24;5(9):e0004763. doi: 10.1371/journal.pgph.0004763 (PMC12459778; doi:10.1371/journal.pgph.0004763)
Supplement: Attachment — Submitted filename: response to reviewers.docx [file pgph.0004763.s004.docx]

Dr Hussein H Twabi
Kamuzu University of Health Sciences
Department of Pathology
[husseintwabi@hotmail.com](mailto:husseintwabi@hotmail.com)

11 July 2025

The Editor
PLOS Global Public Health (PGPH)

Dear Editor,

Response to reviewer comments on the manuscript Ref No. **PGPH-D-25-01300**

Thank you for considering our paper entitled *Acceptability of Self-sampling for High-risk HPV DNA Testing for Primary Cervical Cancer Screening Among Women in Thyolo, Malawi: A Qualitative Study* for publication in your journal. The comments from the reviewers were graciously received and reflect a depth of knowledge and understanding of the topic at hand. Below are responses to those comments, italicized, after the specific comments by each reviewer.

**Responses to Reviewer 1 comments**

Lines 73-75: The authors provide the global number of incident cervical cancer cases and deaths, not the numbers limited to Sub-Saharan Africa.

*Response: The incidence estimates have been updated, including updating the citation.*

Lines 96-98: The authors reference emerging POC HPV tests, referencing an article from 2015. There is still no currently approved POC HPV test, and many countries have already moved toward recommendations of HPV testing in their national strategy. The authors may be referring to lower cost testing that requires less complex laboratory infrastructure or trained personnel.

*Response: The sentence has been amended accordingly.*

Lines 111-118: It’s not clear how self-sampling, which would ostensibly increase the number of HPV tests done, would alleviate the burden on an overextended diagnostic system or address the follow-up challenges still inherent for results provision and appropriate management.

*Response: We acknowledge that self-sampling may lead to an increased number of HPV samples submitted for testing, which in itself could exert pressure on diagnostic capacity. However, our interpretation of reduced burden is situated within the broader operational and service delivery context, where self-sampling alleviates the clinical and logistical demands placed on health workers by reducing the need for provider-led sample collection and associated procedures (e.g., speculum exams). This redistribution of tasks—shifting sample collection from provider to client—has the potential to improve workflow efficiency, particularly in resource-limited settings where healthcare providers are overextended. Moreover, self-sampling facilitates community- or home-based sample collection, minimizing the need for facility visits and enabling task-sharing with community health workers for logistics such as sample transport and patient tracing. While we recognize the limitations of current diagnostic infrastructure, the approach can be scaled with complementary investments in sample transport, decentralised testing hubs, and digital platforms for results dissemination. We have added text in the discussion to clarify this point.*

It’s not clear whether the participants in the study had seen or used the novel device for screening as part of/in addition to the self-sampling for Xpert HPV. Given the complexity of the process (requiring a self-lavage), it would be valuable to understand how women experienced self-collection with that device, in comparison to using a flocked swab. Is that what is meant by “utilized the intervention” which is mentioned several times in the paper?

*Response: Yes, all participants in our study had used the Delphi Vaginal Self-Sampler as part of a broader feasibility study evaluating its integration with near-point-of-care HR-HPV testing (GeneXpert platform). This is the intervention that we refer to. As noted in the Methods section (Lines 141 - 150), women self-collected cervical samples using this device, which employs a lavage-based collection mechanism. As self-sampling has not been introduced to the routine screening channels in Malawi, the participants had no experience with any other self-sampling modalities such as the Floq swab. We acknowledge that this may be a limitation for broader insights into women’s preferences. However, since this was not the primary aim of the study, we believe that our study provides the basis for future exploration on this subject matter.*

What are the screening guidelines for Malawi? In most settings, a 19-yr old, HIV-uninfected, would not be offered screening.

*Response: The Malawi Cervical Cancer Screening Guidelines stipulate that both HIV positive and negative women aged at least 25 should go for cervical cancer screening. However, operationally, younger women are also found to present at the cervical cancer screening clinics. The recommendation from the Sexual and Reproductive Health Directorate was that these women should not be sent back home, as it was observed that it had a negative effect on the screening attendance in all ages. We therefore routinely attend to younger women who present to these clinics. A sentence to elaborate on this has been included in the methods section.*

Table 2: Please adjust the formatting so that there are no “hanging” or wraparound words.

*Response: the table has been amended as per suggestion*

Lines 337-343: What process of integrated self-sampling and self-screening were the IDIs probing/the women describing here?

*Response: The participants’ references to “integrated self-sampling and self-screening” were not based on an actual process offered during the study but rather reflect their aspirations and interpretations during the in-depth interviews. Specifically, some women expressed a desire for a more streamlined and autonomous process whereby they could not only self-collect the sample but also conduct the test and interpret results themselves – essentially mirroring the experience of other self-diagnostic tools like pregnancy or HIV self-tests.*

There are a few limitations that bear mentioning in the limitations section; the authors only spoke with women who had already engaged in self-sampling. This limited their ability to understand barriers to acceptability. In addition, while many studies have shown widespread acceptability of self-screening, the need to ensure appropriate follow-up is crucial, especially in contexts where women have screened outside a health care system or without a provider’s counseling. Did the researchers consider asking about how women would respond to or prefer to receive results or navigate follow-up care?

*Response: The limitations have been expanded to note this. The authors did not probe about women’s preferences about how to receive results or engage in follow up care. However, some women made mention of the fact that self-sampling may facilitate more convenient patient navigation as the women are able to collect the sample themselves then deliver the sample to the hospital. Further enquiry is certainly warranted to tease this out comprehensively.*

**Responses to Reviewer 2 comments**

Background: Additional and recent references on the prevalence and incidence of cervical cancer worldwide and in Malawi should be included. The focus should be on the general population, since the study was conducted among the general population of women and not women living with HIV (Line 81).

*Response: the line about the burden being aggravated by HIV was included to emphasize the fact that the epidemiology of cervical cancer is proportionally higher than most countries, including those with similar socioeconomic levels. We believe this cements the problem and rationalizes why there is need to conduct research and design interventions for cervical cancer control.*

Methods: The methods section should be more structured to enhance readability; the study design, sampling technique, sample size, etc should come first before the intervention. The data management and analysis plan, and ethical considerations should end the section.

*Response: The authors structured the methods section this way to emulate the structure of the COREQ framework for reporting of qualitative studies.*

Provide a justification for the sample size e,g. was saturation attained at the 10th participant? In addition, was a sampling matrix prepared as an inclusion criteria before recruiting study participants?

*Response: The sample size of ten participants was determined based on the qualitative methodological principle of data saturation. We employed a purposive sampling approach to ensure variation in age, educational background, marital status, and HIV status, which are factors known to influence healthcare-seeking behavior in this context. Saturation was considered to be achieved by the 10th interview, as no new themes were emerging and there was noticeable repetition of ideas and perspectives across participants. This was verified through daily debriefings and iterative analysis conducted during data collection. A formal sampling matrix was not developed a priori, but inclusion criteria were clearly defined as: (1) women aged 18 years and above, (2) who had participated in the broader feasibility study, and (3) who had self-sampled using the Delphi Vaginal Self-Sampler for Xpert HPV testing. The purposive selection process was guided by the need to capture a range of experiences rather than representativeness, consistent with the phenomenological design of the study. This rationale has now been clarified in the Methods section of the revised manuscript.*

Also, include information about incentive if any given to the participants. What other permissions and approvals were obtained?

*Response: participants received a participant compensation of $10 as per the guidelines stipulated by the local institutional review board, COMREC. Permission was received from the district health research committee to conduct the study at Thyolo District Hospital.*

The abbreviation “TFA” should be defined at first mention (Line 159).

*Response: the suggestion has been incorporated in the manuscript*

Why was ethicality and opportunity cost excluded from the analysis? (Line 225).

*Response: Ethicality and opportunity cost were excluded from the final analysis because these constructs did not meaningfully emerge during data coding and theme development. Specifically, participants did not spontaneously raise concerns or reflections related to the alignment of the intervention with their personal value systems (ethicality) or the trade-offs involved in engaging with the intervention in place of other valued activities (opportunity cost). As is common in qualitative studies guided by the TFA, not all constructs may be equally salient or relevant across different contexts or populations. Our decision to focus on the five constructs (affective attitude, burden, self-efficacy, intervention coherence, and perceived effectiveness) was based on inductive thematic saturation and relevance to participant narratives. We have included a statement to clarify this in the methods.*

Supporting documents should include responses from the COREQ checklist (Line 234).

*Response: a supplemental file has been added to the manuscript*

Result: The ages of the participants looks like they were randomly selected and not purposive. What was the criteria for the purposive selection of the study participants (Table 2)?

*Response: As indicated, we did not use probability or random sampling methods. Participants were purposively selected from among women who had participated in the self-sampling for cervical cancer screening feasibility study. The purposive sampling aimed to capture variation in age, educational background, marital status, and HIV status – factors known to influence health-seeking behaviour and screening uptake. Our goal was to obtain diverse perspectives on the acceptability of self-sampling, rather than statistical representativeness. We have clarified this in the Methods section.*

Please note that manuscript writing is not a dissertation. Hence, the characteristics of the study participants can be shortened and referred to table 2. Avoid repeating points.

*Response: the results section has been shortened accordingly*

Privacy has been explained in the earlier paragraph. If there is no direct quote for comfort, consider merging privacy and comfort as one (Line 287-294).

*Response: the privacy quote has been moved up but kept nonetheless as it alludes to the positive experiences women experience with regards to privacy brought on by the self-sampling technique, as opposed to the previous paragraph that focuses on the negative experiences of women faced when they are faced with clinician sampling.*

You can focus on the quote directly by excluding “in addition” (Line 303).

*Response: this suggestion has been incorporated in the manuscript*

I suggest that a conceptual framework be drawn to link all the factors in the TFA construct identified in the study.

*Response: a conceptual framework has been drawn and included in the manuscript*

Line 310-311 has already highlighted time as a burden. It is again raised in the next paragraph, Line 320-323.

*Response: the manuscript has been edited to remove the repetition*

Use the past tense of perceive (Line 410).

*Response: this has been corrected as per suggestion*

Discussion: For a more robust discussion, consider including the following references:
1. Bakiewicz A, Rasch V, Mwaiselage J, Linde DS. “the best thing is that you are doing it for yourself” - Perspectives on acceptability and feasibility of HPV self-sampling among cervical cancer screening clients in Tanzania: A qualitative pilot study. BMC Womens Health. 2020;20(1):1–9.
2. Sormani J, Kenfack B, Wisniak A, Datchoua AM, Makajio SL, Schmidt NC, et al. Exploring factors associated with patients who prefer clinician-sampling to HPV self-sampling: A study conducted in a low-resource setting. Int J Environ Res Public Health. 2022;19(1):1–11.
3. Saidu R, Moodley J, Tergas A, Momberg M, Boa R, Wright T, et al. South african women’s perspectives on self-sampling for cervical cancer screening: A mixed-methods study. South African Med J. 2019;109(1):47–52.
4. Pierz AJ, Ajeh R, Fuhngwa N, Nasah J, Dzudie A, Nkeng R, et al. Acceptability of Self-Sampling for Cervical Cancer Screening Among Women Living With HIV and HIV-Negative Women in Limbé, Cameroon. Front Reprod Heal. 2021;2(January):1–8.

*Response: the discussion has been expanded to included discourse that cites some of these papers*

Sincerely,

Hussein H Twabi

Corresponding author

(On behalf of the study team)
