# Supplementary material for: Acceptability of self-sampling for high-risk HPV DNA testing for primary cervical cancer screening among women in Thyolo, Malawi: A qualitative study
Source: PLOS Glob Public Health. 2025 Sep 24;5(9):e0004763. doi: 10.1371/journal.pgph.0004763 (PMC12459778; doi:10.1371/journal.pgph.0004763)
Supplement: Attachment — Submitted filename: response to reviewers 2.docx [file pgph.0004763.s005.docx]

Dr Hussein H Twabi
Kamuzu University of Health Sciences
Department of Pathology
[husseintwabi@hotmail.com](mailto:husseintwabi@hotmail.com)

21 August 2025

The Editor
PLOS Global Public Health (PGPH)

Dear Editor,

Response to second round of reviewer comments on the manuscript Ref No. **PGPH-D-25-01300**

Thank you for considering our paper entitled *Acceptability of Self-sampling for High-risk HPV DNA Testing for Primary Cervical Cancer Screening Among Women in Thyolo, Malawi: A Qualitative Study* for publication in your journal. We appreciate the time given to review this manuscript. Below are responses to those comments, italicized, after the specific comments by each reviewer.

*Journal Requirements:*

**1. If the reviewer comments include a recommendation to cite specific previously published works, please review and evaluate these publications to determine whether they are relevant and should be cited. There is no requirement to cite these works unless the editor has indicated otherwise.**

*The previous reviewer comments included several suggested citations to be included in the discussion. The authors reviewed these and included the citations for the most relevant papers in the discussion section of the manuscript. Otherwise, the rest of the suggestions were omitted.*

**2. Please review your reference list to ensure that it is complete and correct. If you have cited papers that have been retracted, please include the rationale for doing so in the manuscript text, or remove these references and replace them with relevant current references. Any changes to the reference list should be mentioned in the rebuttal letter that accompanies your revised manuscript. If you need to cite a retracted article, indicate the article’s retracted status in the References list and also include a citation and full reference for the retraction notice.**

*We have reviewed all the references included in the manuscript for correctness and completeness. We have not cited any retracted papers. If there is any paper that the editors are having problems accessing, please notify us.*

*Reviewer 1*

**1. In the methods section, the authors need to clearly state that they recruited women who had undergone cervical cancer screening using Xpert HPV with the Delphi Vaginal self-sampler. Both reviewers requested clarification on that, and the revisions to describe purposive sampling and inclusion are not adequate.**

*Thank you for the question. To clarify, the routine process for cervical cancer screening in Malawi does not involve the self-collection of cervical samples using the Delphi lavage sampler. Women were recruited into a broader feasibility study for cervical cancer self-sampling based on whether they had undergone the standard routine cervical cancer screening that includes clinician sampling and Xpert HPV testing. These women were then approached as part of the broader feasibility study for cervical self-sampling. We have included the following to the methods [Line 179-187]: “This study purposively recruited 10 women from a larger feasibility study that was evaluating the use of a novel HPV self-sampling kit coupled with a point-of-care (POC) rapid testing kit for primary HPV screening. Eligible participants were women residing in the catchment area of Thyolo District Hospital who had previously undergone cervical cancer screening using clinician-collected samples tested with Xpert HPV. As part of the feasibility study, each participant was provided with a self-sampling kit, received instruction on how to collect a cervicovaginal sample, and was directed to a private space to perform the collection. The self-collected samples were then submitted to the research team for later batch processing with the POC rapid test.” Additionally, the study setting has been further described from line 133 to line 135 as follows “This study was conducted as part of a broader study investigating the feasibility of a novel HPV self-sampling kit coupled with a POC rapid testing kit for primary HPV testing for cervical cancer screening at Thyolo District Hospital (TDH), a secondary level healthcare facility in Thyolo District in south-western Malawi”.*

**2. In addition, were in-depth interview participants already participants in a larger implementation trial, or had the Delphi Vaginal self-sampler been introduced as standard for self-collection?**

*The in-depth interview participants were already participants in the larger feasibility study. We have clarified this is the methods cited above.*

**3. Overall, the paper lacks a discussion of how women would access follow-up or treatment after self-sampling, as screening alone has no public health benefit without appropriate action for women who screen positive. The expanded discussion paragraph starting on line 524 may be an appropriate place to add this as the authors may be alluding to this, and/or in the limitations or conclusion paragraph.**

*Thank you for the question. As it stands, the paper aims to demonstrate the utility of self-sampling in improving uptake of cervical cancer screening in Malawi, without focusing on the outcomes of an abnormal result on screening, as we believe health seeking behaviour is generally better in individuals who know they have a pathological condition as opposed to those who do not know. Nevertheless, we agree with the views of the reviewer and have added a sentence to the suggested paragraph to propose an approach to linkage to care once results are out. “Furthermore, it is equally important to establish clear referral pathways to ensure that women with abnormal results are promptly linked to confirmatory testing and treatment, possibly through similar integration with existing community health structures and follow-up support by HSAs.”*

*Reviewer 2*

**Just a slight correction for introductory part of the manuscript; Line 70-84: Use the funnel approach; worldwide, sub-Saharan Africa, and then Malawi.**

*Thank you for this suggestion. As it stands, we believe we have observed the funnel approach as we begin by talking about the worldwide epidemiology and raking of cervical cancer, followed by mention of the estimates for SSA, followed by the epidemiology of the disease in Malawi, as demonstrated by the color coded excerpt that follows: “Cervical cancer ranks as the fourth most prominent cause of female cancer cases worldwide, contributing significantly to cancer-related fatalities in women.[1] It is particularly devastating in low- and middle-income countries (LMICs), with sub-Saharan Africa bearing the highest burden.[1] In sub-Saharan Africa in 2020, there were approximately 117,944incident cases of cervical cancer, along with 76,140associated deaths, which translates to standardised rates of 33.4 new cases and 22.6 deaths per 100,000 individuals, respectively.[1]*

*Malawi stands out as having the world's highest recorded rates of cervical cancer incidence and mortality. With an age-standardised incidence rate of 67.9 per 100,000 and an age-standardised mortality rate of 51.5 per 100,000, the country’s burden of disease is double the sub-Saharan African estimates.[2] Additionally, there is a rising trend in the number of new cervical cancer cases in the country.[3] The burden of cervical cancer in Malawi is further aggravated by its association with HIV, as the disease is strongly linked to the prevalence of HIV regardless of antiretroviral therapy.[4–6]”*

**Also, incentives given to participants and permissions received before conducting the study should be included in the ethical consideration section of the manuscript.**

*Thank you for this comment. Line 254 – 255 “The district Health Research Committee provided permission to conduct the study at Thyolo District Hospital” addresses the comment about the permissions. According to local IRB regulations, participants are to be compensated 10USD for participation in health research. This compensation was a total amount and covered time for both the larger feasibility study and this qualitative study. We have included the line “In accordance with COMREC regulations, all participants received $10 as compensation for their time given to broader feasibility study as well as this qualitative component”.*

Sincerely,

Hussein H Twabi
Corresponding author
(On behalf of the study team)
